# Aenigmachannidae, a new family of snakehead fishes (Teleostei: Channoidei) from subterranean waters of South India

**DOI:** 10.1038/s41598-020-73129-6

**Published:** 2020-09-30

**Authors:** Ralf Britz, Neelesh Dahanukar, V. K. Anoop, Siby Philip, Brett Clark, Rajeev Raghavan, Lukas Rüber

**Affiliations:** 1 Museum of Zoology, Senckenberg Natural History Collections Dresden, E01109 Dresden, Germany; 2 Department of Life Sciences, Natural History Museum, London, SW75BD UK; 3 Indian Institute of Science Education and Research (IISER), Dr. Homi Bhabha Road, Pashan, Pune, 411 008 India; 4 Zoo Outreach Organization, No. 12 Thiruvannamalai Nagar, Saravanampatti - Kalapatti Road, Coimbatore, 641 035 India; 5 School of Ocean Science and Technology, Kerala University of Fisheries and Ocean Studies (KUFOS), Kochi, 682 506 India; 6 Department of Zoology, Nirmalagiri College, Kannur, India; 7 Imaging and Analysis Centre, The Natural History Museum, London, SW7 5BD UK; 8 Department of Fisheries Resource Management, Kerala University of Fisheries and Ocean Studies (KUFOS), Kochi, 682 506 India; 9 Naturhistorisches Museum Bern, 3005 Bern, Switzerland; 10 Aquatic Ecology and Evolution, Institute of Ecology and Evolution, University of Bern, 3012 Bern, Switzerland

**Keywords:** Evolution, Zoology

## Abstract

Pronounced organism-wide morphological stasis in evolution has resulted in taxa with unusually high numbers of primitive characters. These ‘living fossils’ hold a prominent role for our understanding of the diversification of the group in question. Here we provide the first detailed osteological analysis of *Aenigmachanna gollum* based on high-resolution nano-CT scans and one cleared and stained specimen of this recently described snakehead fish from subterranean waters of Kerala in South India. In addition to a number of derived and unique features, *Aenigmachanna* has several characters that exhibit putatively primitive conditions not encountered in the family Channidae. Our morphological analysis provides evidence for the phylogenetic position of *Aenigmachanna* as the sister group to Channidae. Molecular analyses further emphasize the uniqueness of *Aenigmachanna* and indicate that it is a separate lineage of snakeheads, estimated to have split from its sister group at least 34 or 109 million years ago depending on the fossil calibration employed. This may indicate that *Aenigmachanna* is a Gondwanan lineage, which has survived break-up of the supercontinent, with India separating from Africa at around 120 mya. The surprising morphological disparity of *Aenigmachanna* from members of the Channidae lead us to erect a new family of snakehead fishes, Aenigmachannidae, sister group to Channidae, to accommodate these unique snakehead fishes.

## Introduction

Among the seemingly endless diversity of animal life on our planet, a few extant species hold a unique position for our understanding of the evolution of the group to which they belong. Such taxa have previously been characterized with the term ‘living fossil’ starting with Darwin^1^ or have been referred to as ‘basal taxa’^2^. They exhibit a striking level of morphological stasis as evidenced by a surprisingly large number of primitive characters compared to their extant sister group, and often represent lineages with only few extant representatives and a restricted distribution. Widely known examples of ‘living fossils’ or ‘basal taxa’ among vertebrates are the coelacanth *Latimeria*, the lungfish *Neoceratodus*, the tuatara *Sphenodon* or the platypus *Ornithorhynchus*. One of the most recent vertebrate examples is the eel *Protanguilla palau*^3^, which combines many primitive morphological characters otherwise only known from Cretaceous fossil eels.

As we show here, *Aenigmachanna gollum* from subterranean waters in the Western Ghats area of Peninsular India is another recent discovery that fits the characterisation of ‘basal taxon’^2^. The area where *Aenigmachanna* was collected is part of the Western Ghats – Sri Lanka Hotspot, one of the most significant global biodiversity hotspots^4–6^. Its high levels of endemism^6–8^ are not only the result of recent radiations^9^, but also of the presence of a number of ancient lineages^10–12^. Especially among vertebrates there are a number of such relic lineages in the Western Ghats, often with unclear phylogenetic relationships, such as the cyprinid *Lepidopygopsis*^13^ and the catfish *Kryptoglanis*^14^, or with remote biogeographical connections, such as the burrowing frog *Nasikabatrachus*^15^.

Some of the strangest relic lineages among the vertebrates of the Western Ghats are freshwater fishes from subterranean waters in the laterite areas at the foothills along the coast. A total of ten freshwater fish species have been described from this unusual habitat since the mid-1900s: three species of the blind, pigment-less catfish genus *Horaglanis*^16–18^, the enigmatic catfish *Kryptoglanis*^19^, three highly elongate, whip-like species of the swamp eel genus *Monopterus*^20–22^, the miniature, dorsal- and pelvic-finless eel loach *Pangio bhujia*^23^ and the recently discovered Gollum snakehead *Aenigmachanna gollum*^24^ as well as its sister species *A. mahabali*^25^.

Snakehead fishes are freshwater predators of medium to large size distributed in West and Central Africa and large parts of Asia^26^. They are obligatory air breathers that swallow air into their suprabranchial organ, in which gas exchange takes place allowing them to survive in oxygen-deprived waters and even on land^27^. Some species have become highly invasive outside of their native range and two species are now established in the United States^28,29^. Snakehead fishes have parental care and either primitively guard a nest of floating eggs at the water surface or, as a more derived condition, are mouthbrooders^30^.

Here, we provide the results of the first osteological study on the subterranean snakehead fish *Aenigmachanna* obtained from high resolution nano-CT scans. In addition, we performed the first morphological and molecular analysis aimed at exploring its phylogenetic position among Channoidei. We show that *Aenigmachanna* has an unexpectedly large number of primitive conditions in its skeleton in relation to its sister group the Channidae. The uniqueness of *Aenigmachanna* is further supported by our molecular analysis, which also indicates a surprisingly old age for this lineage. Based on our morphological and molecular results we place *Aenigmachanna*, comprising two species, into its own family, Aenigmachannidae.

## Results and discussion

### Taxonomy

**Figure 1:**
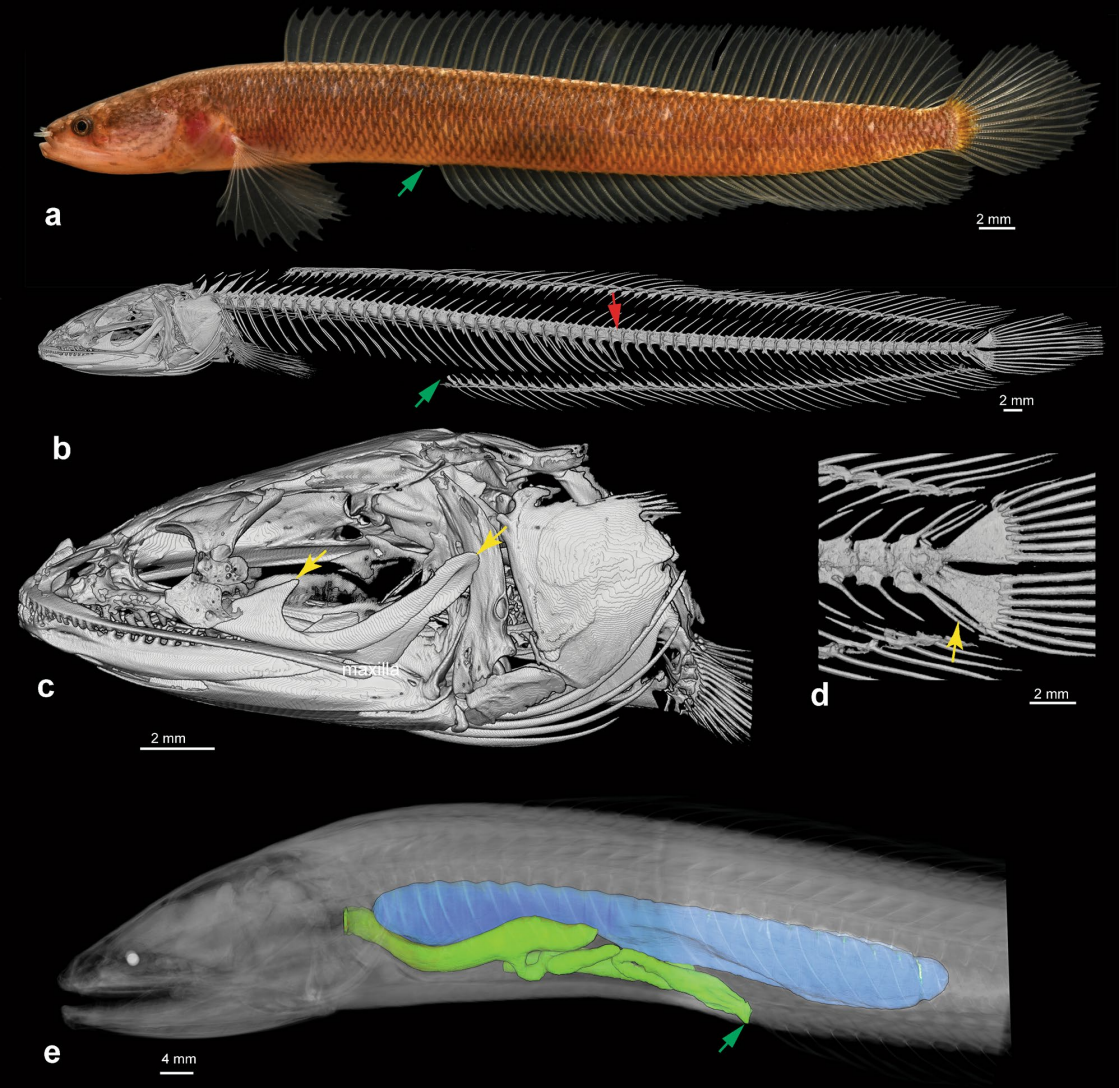
*Aenigmachanna gollum*. (**a**) 60.6 mm individual alive (KUFOS 2019.8.226), green arrow marks position of vent; (**b**) CT scan image of skeleton of 90.2 mm holotype (BNHS FWF 966), green arrow marks position of vent and red arrow the first caudal vertebra; (**c**) CT scan image of head of holotype, yellow arrows mark postorbital process and posterior tip of greatly elongated maxilla; (**d**) CT scan image of caudal skeleton of holotype, note absence of Day’s bone and presence of distally bifurcated haemal spine (yellow arrow) on second preural centrum; (**e**) CT scan image of iodine stained 124.5 mm specimen (KUFOS 2019.8.225) in lateral view, swim bladder is shown in blue above the digestive system (green), note swim bladder ending at level of 8th postanal vertebra.

**Figure 2:**
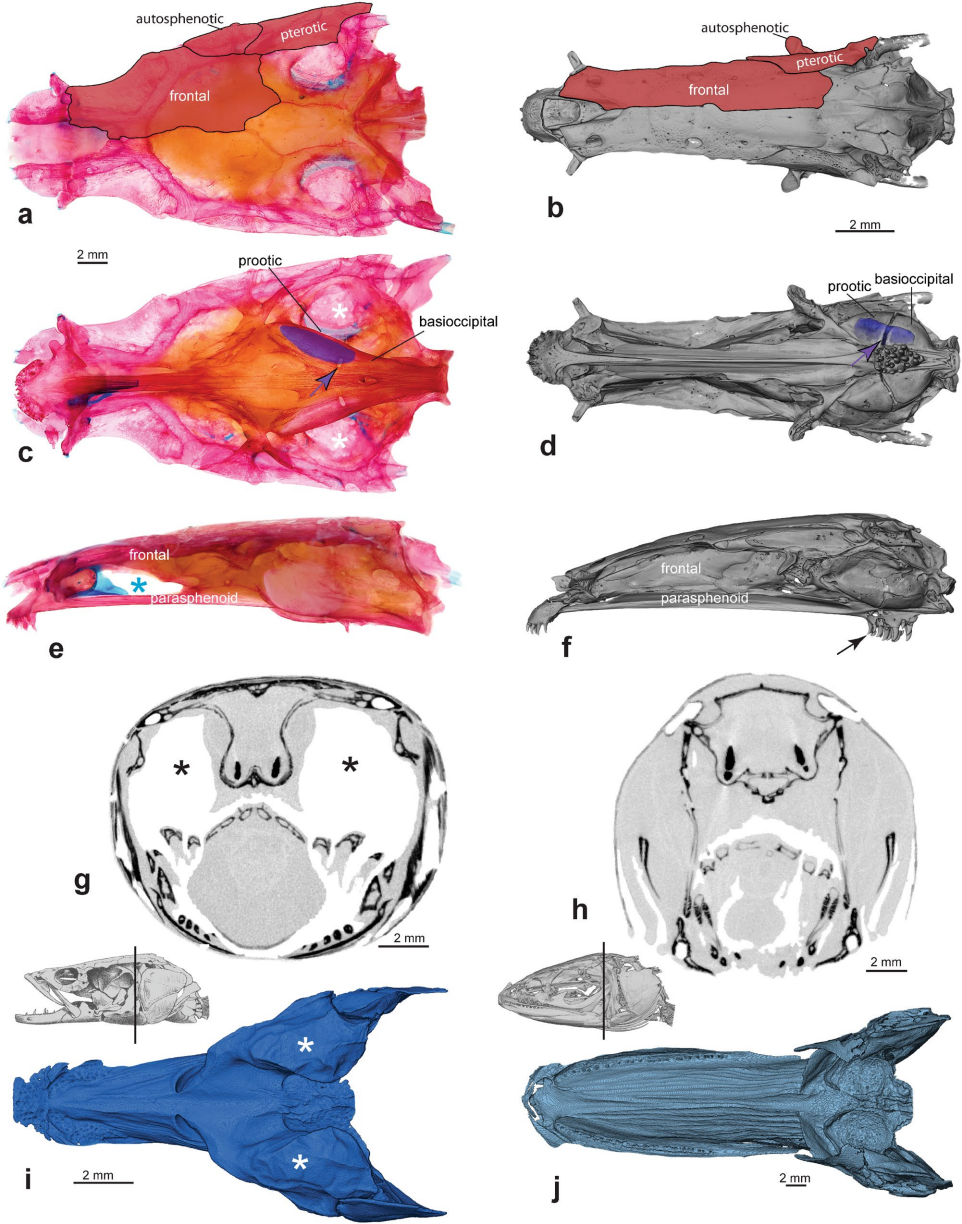
Head anatomy of a channid (*Parachanna*, left side) and *Aenigmachanna* (right side) in comparison. Neurocranium of *Parachanna africana*, (MTD-F39824, c&s, nasals not removed) 150 mm (**a**,**c**,**e**) and *Aenigmachanna gollum*, (KUFOS 2019.8.224) 81.8 mm in dorsal (**a**,**b**), lateral (**c**,**d**) and ventral (**e**,**f**) view. *Parachanna* (**a**) with broad contact between frontal and autosphenotic but *Aenigmachanna* (**b**) with broad contact of frontal and pterotic excluding autosphenotic from skull roof. *Parachanna* with wide skull forming roof (marked with white stars in (**c**) over suprabranchial cavity, sacculith shown in purple and prootic-basioccipital suture of otic capsule marked by purple arrow. Note presence of large parasphenoid tooth patch and complete interorbital septum formed by contact of frontal and parasphenoid in *Aenigmachanna* (**f**) and presence of interorbital fenestra (marked with blue *) in *Parachanna* (**e**). Transverse CT sections through the head of *Parachanna obscura*, (CUMV 96491), length unknown, (**g**), and *Aenigmachanna*, 124.5 mm (KUFOS 2019.8.224), [vertical lines in (**g**,**h**) show level of section] to illustrate presence [marked by black stars in (**g**)] or absence of large suprabranchial cavity. Virtual casts of internal space of mouth and branchial cavity confirm presence of large paired suprabranchial cavities in *Parachanna* (**i**) and their absence in *Aenigmachanna* (**j**).

Aenigmachannidae (Gollum snakehead fishes), new family; type genus *Aenigmachanna* Britz, Anoop, Dahanukar & Raghavan 2019 (Figs. 1, 2).

**Figure 3:**
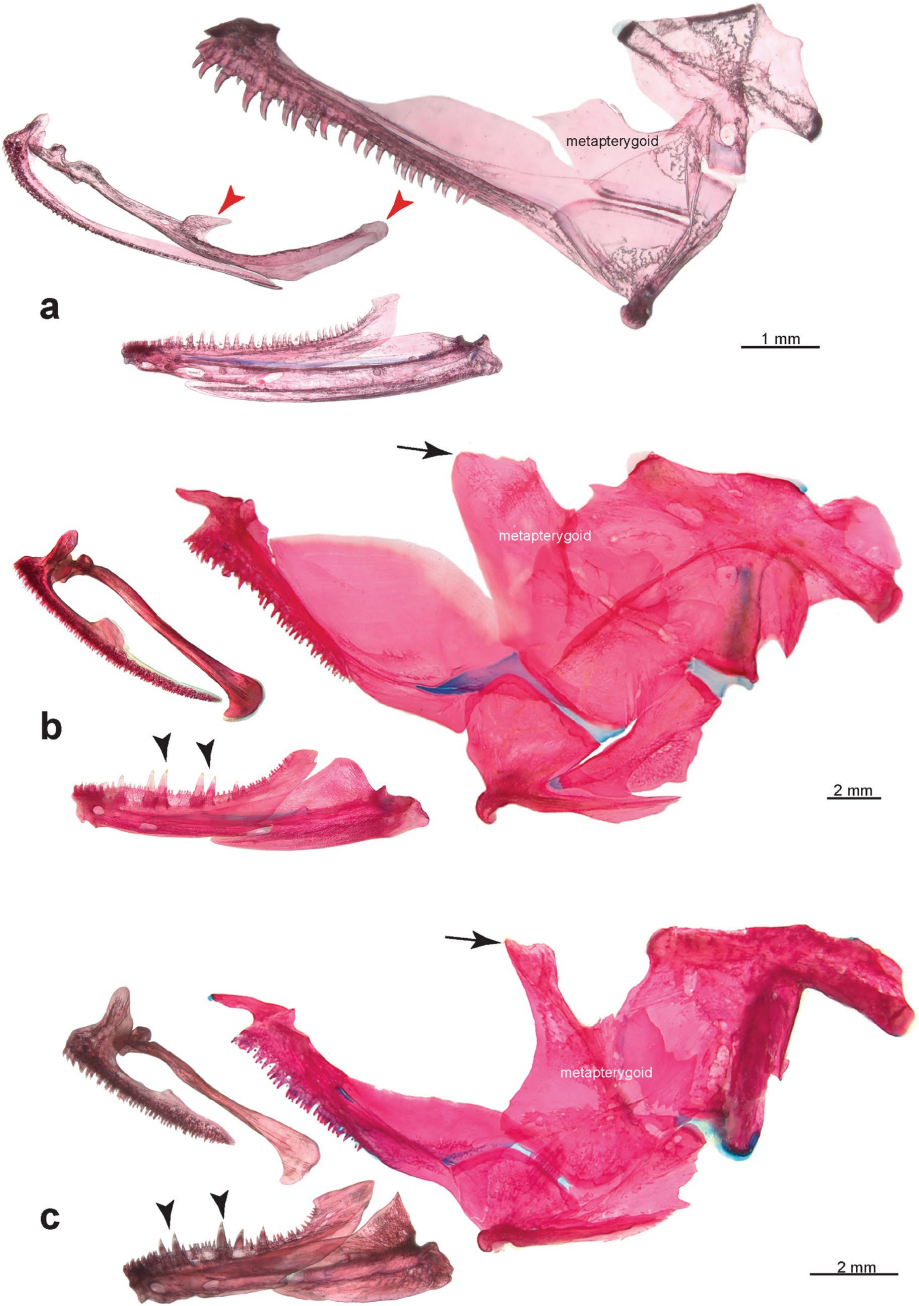
Cleared and stained jaws and hyopalatine arch in lateral view of (**a**) *Aenigmachanna gollum*, (KUFOS 2019.8.226) 60.6 mm, (**b**) *Parachanna africana*, (MTD-F39824) 150 mm and (**c**) *Channa punctata*, (MTD-F39825) 118 mm. Note presence of maxillary processes (marked by red arrowheads) in (**a**) and their absence in (**b**) and (**c**), presence of caniniform teeth (marked by black arrowheads) in dentary in (**b**) and (**c**) and their absence in (**a**) and presence of long uncinate process (marked by black arrows) on metapterygoid in (**b**) and (**c**) and its absence in (**a**).

Diagnosis: A family of the acanthomorph clade Labyrinthici (Anabantiformes), as evidenced by the shared derived possession of a parasphenoid tooth patch (Fig. 2d,f). Aenigmachannidae are distinguished from all other Labyrinthici by the following autapomorphies: (a) a very long maxilla reaching caudally beyond the anterior margin of the preopercle (Figs. 1b,c, 3a), (b) presence of a prominent postorbital process on the maxilla (Figs. 1c, 3a), (c) the frontal suturing with the parasphenoid forming a complete interorbital septum (Fig. 2f), (d) the unique count of 29–32 abdominal and 29–31 caudal vertebrae (Fig. 1b), (e) a series of five median predorsal bones (supraneurals or rayless pterygiophores) in front of the dorsal fin (f) 83–85 scales in a lateral series, and (g) a high number of 40–44 anal-fin rays (Fig. 1a,b). It differs further from all Anabantoidei and Channidae by the swim bladder being short (Fig. 1e), not reaching the parhypural and by the absence of a suprabranchial cavity and organ (Fig. 2h,j). Aenigmachannidae share with Channidae long nasal tubes (Fig. 1a), cycloid scales, the absence of fin spines in dorsal- and anal-fins (Fig. 1a), an increase in the number of vertebrae, a single posterior swimbladder extension combined with abdominalisation of the anterior ten postanal vertebrae (Fig. 1), and five branchiostegal rays, but differ from them by the autosphenotic being excluded from the skull roof and the frontal broadly sutured only to the pterotic (Fig. 2a–f), prootic and basioccipital forming equal parts of the bulla for the sacculith (Fig. 2b,c), the presence of numerous caudal vertebrae and therefore the lack of an abdominalisation of the posterior vertebral column (Fig. 1b), and by absence of the uncinate process of the metapterygoid (Fig. 3a), absence of Day’s bone (Fig. 1d), and of a body lateral-line canal.

### Aenigmachannidae: a morphologically plesiomorphic lineage of snakehead fishes

With its elongate body, long and spine-less dorsal and anal fins and anterior nares conspicuously extended as nasal tubes (Fig. 1a) *Aenigmachanna* closely resembles species of the family Channidae in these external features. However, a detailed study of its internal morphological characters reveals a large number of significant differences. Many of these represent a more primitive condition than encountered in any of the species of channids, as revealed by our morphological analysis (Fig. 5, Supplementary Figs. S1, S2) and discussed below. Monophyly of the family Channidae has previously been based on several putative synapomorphies^31,32^ for which we have examined the character state in *Aenigmachanna* (number in parentheses behind character refers to character number in Supplementary Material).

#### a. Otic bulla for sacculith mostly contained in prootic (21)

In *Aenigmachanna* the prootic part of the bulla is not increased in size (Fig. 2d,f) and it houses only about half of the sacculith (Fig. 2d). This is the condition found in all other non-channid members of the Labyrinthici and represents a primitive character state.

#### b. Metapterygoid with anterodorsal uncinate process approaching neurocranium or articulating with it (33)

*Aenigmachanna* lacks the uncinate process of the metapterygoid of channids (Fig. 3). Such a process is also absent from all non-channid Labyrinthici and generally in teleosts and thus represents another primitive condition in *Aenigmachanna*.

#### c. Accessory breathing organs with respiratory nodules on first and second epibranchials, hyomandibular, and parasphenoid (30)

*Aenigmachanna* lacks a suprabranchial cavity and a suprabranchial organ altogether (Fig. 2g–j) and there are no respiratory nodules on epibranchials, hyomandibular or parasphenoid, again a more primitive character state than in Channidae. The lack of a suprabranchial cavity and labyrinth organ in *Aenigmachanna* may indicate that the suprabranchial cavities and organs of channids and anabantoids evolved independently or that *Aenigmachanna* has lost them secondarily. A detailed study of the branchial blood vessels in *Aenigmachanna*, which share synapomorphic conditions in channids and anabantoids, may provide evidence to decide between these two different hypotheses.

#### d. Presence of ‘Day’s bone’ sensu^31^, an autogenous, elongate ossicle (like a detached haemal spine) between haemal spines of PU2 and PU3 (15)

Day’s bone is absent in *Aenigmachanna* (Fig. 1d), which, however, has a bifurcated haemal spine on preural centrum 2. In terms of its position, the anterior part of this bifurcation in *Aenigmachanna* is in the position of Day’s bone in Channidae and may represent a more primitive condition, in which it is not yet autogenous, unless it is fused ontogenetically in *Aenigmachanna* to the haemal spine. This may mean that Day’s bone in channids potentially evolved from a detached anterior portion of a bifurcating haemal spine on preural centrum 2.

#### e. Absence of fin spines in all fins (3,5,8)

*Aenigmachanna* shares with Channidae the derived absence of dorsal- and anal-fin spines, which is combined in both taxa with very long, soft-rayed dorsal (> 30 rays) and anal (> 20 rays) fins (Fig. 1a). Pelvic fins, which are also spineless in channids are absent in *Aenigmachanna*.

#### f. Presence of inner row of caniniform teeth and outer row of very small teeth along length of bone (37)

Murray^32^ added this character as a further putative synapomorphy of Channidae (illustrated here in Fig. 3b,c). *Aenigmachanna* lacks the larger caniniform teeth of channids in its dentary (compare Fig. 3a with b and c), but has a series of conical teeth of similar size (Figs. 1c, 3a), as other non-channid Labyrinthici, again a more primitive condition.

We conclude that *Aenigmachanna* does not share five of the six channid synapomorphies^31,32^, but rather has a more primitive character state compared to that in Channidae.

To further evaluate the phylogenetic position of *Aenigmachanna* in relation to Channidae and other Labyrinthici we performed a parsimony analysis of 46 morphological characters and 15 taxa of Labyrinthici the results of which are shown in Fig. 5. *Aenigmachanna* is recovered as the sister group of the family Channidae a position highlighted by a number of additional primitive characters that *Aenigmachanna* shares with non-channid Labyrinthici, as follows.

#### g. Presence of numerous caudal vertebrae (11)

**Figure 4:**
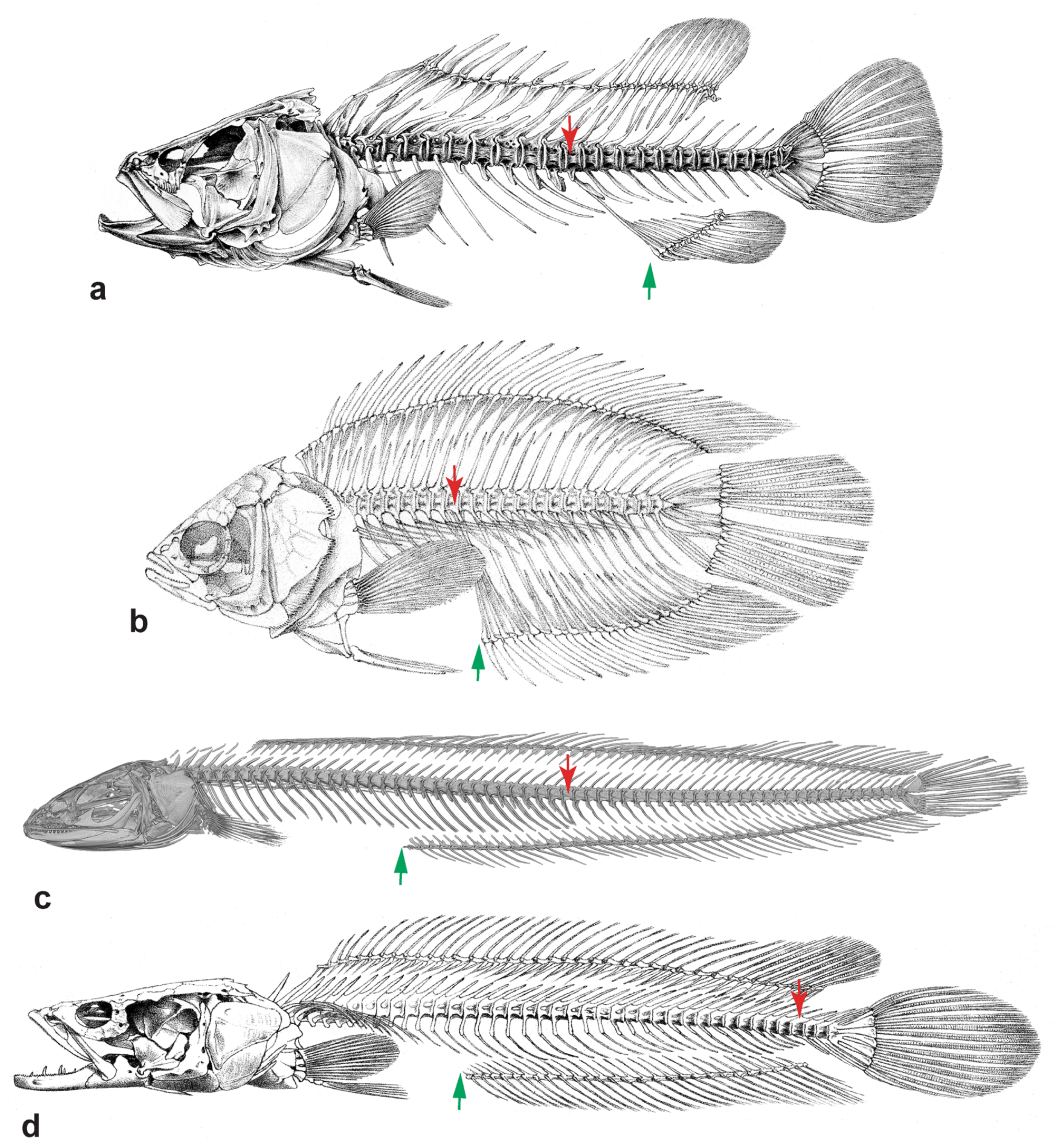
Lateral views of skeleton of the generalized percomorph *Lates* (**a**), the anabantoid *Ctenopoma* (**b**), the aenigmachannid *Aenigmachanna* (**c**) and the channid *Parachanna* (**d**). Green arrow marks position of vent, red arrow position of first caudal vertebra (anterior most vertebra with haemal spine). Note more or less equal separation of vertebral column in *Lates* (**a**) and *Ctenopoma* (**b**) into abdominal and caudal vertebrae (**a**,**b**,**d** adapted from^33^) with position of vent and first caudal vertebra close to each other and caudal and postanal region of vertebral column of similar length. Note partial abdominalisation of postanal vertebral column in *Aenigmachanna* and almost complete abdominalisation in *Parachanna*.

Species of the genera *Parachanna* and *Channa* are highly unusual among teleosts in having elongate bodies with 40–66 vertebrae, of which, however, only the posterior most 4–6 morphologically are caudal vertebrae with haemal spines (Fig. 4). This is combined with the presence of parapophyses and ribs on all vertebrae anterior to these 4–6 true caudal vertebrae. Such an abdominalisation of most of the postanal vertebral column is a synapomorphy of Channidae. Members of their sister group, the Anabantoidei, and indeed all other Labyrinthici, lack this abdominalisation of the posterior caudal region and have a typical separation of the abdominal from the caudal vertebral column roughly in the middle of the body (Fig. 4). *Aenigmachanna* (Figs. 1b, 4) and all non-channid Labyrinthici have the primitive condition of a vertebral column divided into abdominal and caudal sections of roughly similar length (29–32 abdominal and 29–31 caudal vertebrae in *Aenigmachanna*). The posterior 10–12 abdominal vertebrae in *Aenigmachanna* are, however, situated posterior to the anus and anal-fin origin (Figs. 1, 4) but have parapophyses and ribs and lack haemal spines and therefore represent abdominalized vertebrae of the anterior part of the postanal vertebral column. Vertebrae in the same position to the anus/anal fin origin in non-channid Labyrinthici and in generalized percomorphs are caudal vertebrae (Fig. 4).

#### h. Single swim bladder not reaching posteriorly to parhypural, but ending at middle of body (17)

Associated with the abdominalisation of the caudal vertebral column in Channoidei is a posteriorly extended swim bladder that reaches all the way to the parhypural in *Channa* and *Parachanna*^34,35^. A swim bladder extension of similar length is also present in Anabantoidei, but here the elongation comprises paired extensions that run posteriorly along the left and right side of the haemal spines of the caudal vertebrae^34^. A posterior swim bladder extension up to the parhypural was hypothesized as a channoid-anabantoid synapomorphy^31^. The presence of a shorter swim bladder in *Aenigmachanna* (Fig. 1e) that ends at the level of the 8th postanal vertebra sets this taxon apart from all other channoids and anabantoids. This character state is interpreted as a primitive condition, shared with nandid, badid and pristolepidid Labyrinthici and other percomorphs, with the posterior swim bladder elongation having evolved independently in anabantoids and channoids. Such an interpretation may also explain the difference in the nature of the elongation (single midline extension in channids, paired diverticula in anabantoids).

#### i. Autosphenotic is not part of the skull roof and does not carry a lateral line canal (19,20)

Channids are unusual among teleosts in that the temporal canal, usually restricted to dermal bones frontal and pterotic, runs in the autosphenotic, a chondral bone, which also forms part of the skull roof (Fig. 2a). In *Aenigmachanna* and other non-channid Labyrinthici the autosphenotic is excluded from the skull roof and the temporal lateral-line canal runs exclusively in the pterotic and the frontal, which are in direct contact (Fig. 2b).

#### j. Basioccipital lacks paired flanges or processes (22)

Channids have a pair of elongate lateral flanges or round condyles, which form an articulation with the upper pharyngeal jaws via an articular surface on pharyngobranchials 2 + 3. *Aenigmachanna* has no basioccipital flanges or condyles and the upper pharyngeal jaws do not articulate with the basicoccipital. This is potentially another plesiomorphic condition in *Aenigmachanna*. The absence of such processes may also be interpreted as a secondary loss in *Aenigmachanna*, because anabantoids have paired basioccipital pharyngeal processes, which may be homologous to the paired processes in channids. All the above hypothesized primitive character states in *Aenigmachanna* and their apomorphic condition in Channidae suggest that they are sister groups and that Aenigmachannidae represent a lineage that diverged from the other snakehead fishes before these split into the African genus *Parachanna* and the Asian genus *Channa*. Among Labyrinthici, Channidae and Aenigmachannidae share the derived states of the following characters, as putative channoid synapomorphies (see characters 1–5, 7, 9, 10,16, 41 in Supplementary File): long nasal tubes, cycloid scales, absence of dorsal- and anal-fin spines, a high number of dorsal- and anal-fin rays, increase in the number of vertebrae and a single posterior swimbladder extension combined with abdominalisation of at least the anterior postanal vertebral column, and only five branchiostegal rays.

### Aenigmachannidae as a separate lineage of snakehead fishes: molecular divergence time estimates and biogeographical implications

Our analyses of two different molecular datasets, of which one is based on complete mitochondrial genomes, support the view that *Aenigmachanna gollum* forms a lineage, separated from *Channa* and *Parachanna* for a long time. Depending on the dataset we analysed (Supplementary Figs. S3, S4), *Aenigmachanna* is resolved either as the sister group of Channidae (dataset 2), or the sister group of *Channa* (dataset 1). These alternative placements in addition to the alternative position of *Aenigmachanna* as the sister group to *Parachanna*, however, could not be rejected statistically (Supplementary Fig. S3), indicating that the trichotomy at the root of the tree cannot be resolved further with these datasets. Some short internodes located deep in a phylogeny are notoriously difficult to resolve even when genome-scale datasets are applied^36,37^. Here, contentious phylogenetic relationships can be driven even by only a handful of genes, thus hampering our quest to resolve the countless branches of the tree of life^38^. Although one of our datasets was based on complete mitochondrial DNA, only more data on a phylogenomic scale may help to better resolve the topological uncertainty regarding the phylogenetic position of *Aenigmachanna*. Notwithstanding this level of uncertainty, the results of both analyses, emphasize the uniqueness of the *Aenigmachanna* lineage and its long separation from the *Parachanna* and *Channa* lineages.

**Figure 5:**
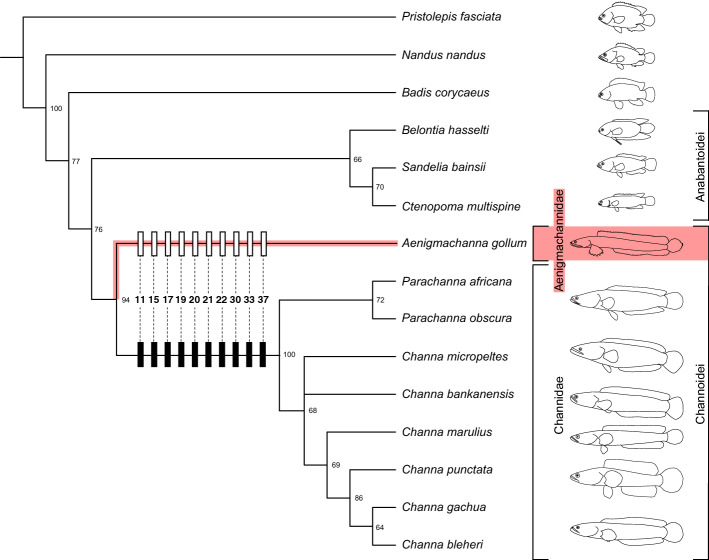
Phylogenetic relationships of Aenigmachannidae (marked in red) based on 46 morphological characters. Shown is the strict consensus tree of two most parsimonious trees (tree length = 64 steps, consistency index (CI) = 0.72, retention index (RI) = 0.89). Numbers at nodes correspond to bootstrap values (1000 replicates). Plesiomorphic character states of *Aenigmachanna* in relation to Channidae discussed in the text are shown on the tree with corresponding numbers.

**Figure 6:**
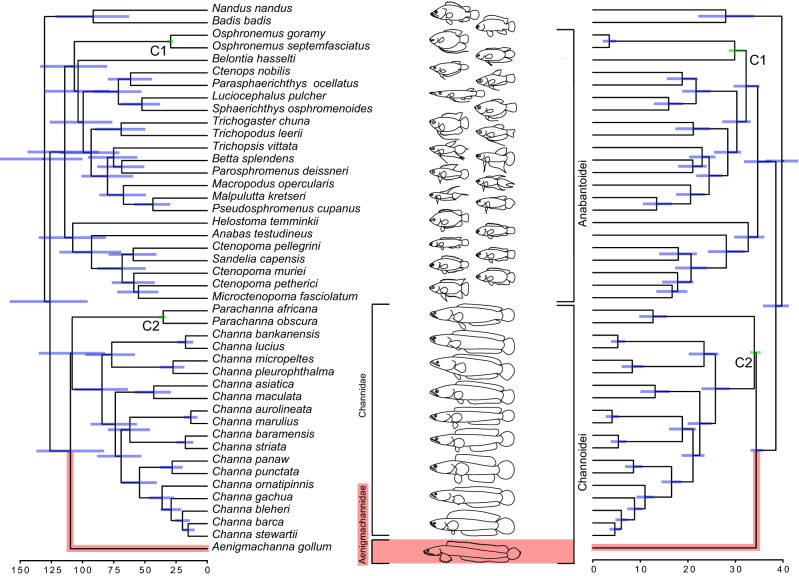
Divergence time estimates for Aenigmachannidae (marked in red) and other Labyrinthici based on molecular data using a constrained topology matching the morphological topology. Shown are crown group (left) and stem group (right) calibration of fossils (C1: ^†^*Osphronemus goramy*; C2: ^†^*Parachanna fayumensis*).

However, our morphological analysis unequivocally places *Aenigmachanna* as the sister group to the Channidae (Fig. 5), which is also highlighted by the large number of morphologically primitive characters of *Aenigmachanna* in relation to Channidae (Fig. 5). Given that this placement of *Aenigmachanna* is further supported by the results of the analysis of dataset 2, and not rejected by dataset 1, we used a constrained tree based on dataset 1, reflecting the sister group relationship of the two taxa, to perform a divergence time estimation (Fig. 6). We chose dataset 1 over dataset 2, as the former allowed us to perform stem and crown based calibrations of the two key fossils that we used. We calculated that *Aenigmachanna* split from its sister group, the Channidae, at least around 109 mya (range 83–136 mya) when the crown calibration is used and 34 mya (range 33–35 mya) with the stem calibration (Fig. 6).

The unique lineage of Aenigmachannidae may also hold a key position among Channoidei for our understanding of snakehead biogeography and evolutionary diversification given its estimated old age. Applying the crown calibration, the range of our minimum age estimate of 83–136 million years for the *Aenigmachanna* lineage takes it back to the period when the Gondwana supercontinent started to break up and India/Madagascar separated from Africa at around 120 million years ago. This would indicate that the presence of *Aenigmachanna* and *Channa* in Asia and *Parachanna* in Africa is the result of vicariance, as recently hypothesized for the Channidae^30^. With the alternative stem calibration and the resulting minimum age of 33–35 million years, a Gondwana vicariance scenario appears less likely, though is still possible. The alternative of dispersal from Africa to India or vice versa after closure of the Tethys can be ruled out as the oldest channid fossil, the African ^†^*Parachanna fayumensis*, predates this closure and formation of land bridges between the Asian and African continents. The third alternative to explain the presence of members of the Channoidei in both Africa and Asia is long distance transoceanic dispersal. However, there are strong biological arguments that contradict such a scenario. In addition to channids being primary freshwater fishes, species of the genus *Parachanna* as well as all basal lineages of the genus *Channa* have well-developed parental care, in which a float of buoyant eggs is guarded at the surface of the water; the derived mouthbrooding has only evolved in the Gachua group within *Channa*. For parsimony reasons this floating egg guarding would have to be hypothesized to represent the primitive condition of reproduction for Channidae as a whole. If one accepts this, then dispersal requiring numerous generations of reproducing adults having to live in marine waters with this kind of reproductive behaviour appears impossible, and parental care involving floating eggs is also unknown among any of the marine teleost species. This leaves as the most plausible scenario to explain channoid African Asian distribution a Gondwanan vicariance, in which the lineages of *Aenigmachanna* and *Channa* were present on a northward drifting Indian plate.

The existence of such an old, unique lineage that has conserved a large number of plesiomorphic conditions in relation to its more species rich sister group is not uncommon among fishes. Stiassny & de Pinna^2^ provided examples from freshwater fishes of what they termed ‘basal taxa’, i.e. phylogenetically and morphologically primitive taxa that have a unique significance for the understanding of the evolution of the often species-rich sister group. They also noticed that these ‘basal taxa’ frequently have a restricted distribution, which makes them highly susceptible to habitat alterations and environmental pressures. This also applies to *Aenigmachanna*, the subterranean habitat of which is greatly influenced by changes in groundwater levels in the densely populated parts of the coastal laterite areas with more than 6 million homestead wells tapping into the state’s aquifers^39^.

The discovery of new fish species, such as *Aenigmachanna gollum* and *A. mahabali*, that cannot be clearly assigned to known family-level taxa is rare, but in the last eight years, three new teleost families, all monotypic, have been described to accommodate morphologically highly unusual bony fishes: the anguilliform *Protanguilla palau*^3^, the catfish *Kryptoglanis shajii*^14^ and the characiform *Tarumania walkerae*^40^. Along with these three taxa, *Aenigmachanna* is, in our opinion, one of the most interesting ichthyological discoveries of the last decade, and further detailed studies are needed to fully understand its anatomical characteristics and basic features of its biology, especially its reproduction, as well as its habitat requirements and distribution in South India.

## Methods

### Morphology

The holotype (BNHS [Museum of the Bombay Natural History Society] FWF 966) and paratype (BNHS FWF 967) of *Aenigmachanna gollum* and two additional specimens, 81.8 mm standard length [SL] (KUFOS [Kerala University of Fisheries and Ocean Sciences] 2019.8.224) and 124.5 mm SL (KUFOS2019.8.225) were scanned with a Zeiss Versa Nano CT scanner. The 124.5 mm specimen was stained in a 5% iodine solution for four days before scanning. Depending on the specimen (stained or unstained) a voltage of 60 to 90 kV was selected to optimize X-ray transmission. A rotation of 360 degrees was used collecting 1601 projections for each scan. Using both geometric and optical magnification (0.4 × lens) techniques along with the vertical stitching function, a final voxel size of 16.6 μm was possible for the holotype and paratype. A similar approach resulted in a voxel size of 15.1 μm for the 124.5 mm (iodine-stained) specimen. With the 81.8 mm specimen we focused on attaining a high pixel resolution of the skull with a 5.7 μm voxel size. Data reconstruction was implemented using the Zeiss Reconstructor Scout-and-Scan software. A filtered back projection algorithm and beam hardening correction was used to reconstruct the projections to produce a stack of 16-bit tiff images.

For comparison, a fifth scan of the channid *Parachanna obscura* (Cornell University Fish Collection CUMV 96491) was downloaded from MorphoSource (https://www.morphosource.org/Detail/MediaDetail/Show/media_id/33518).

All 3D rendering was made using the Avizo (2019.1) software package. A median filter was used to reduce image noise to allow for accurate segmentation. Region growing tools such as the magic wand were used to segment individual bones slice by slice. Individual or series of bones were masked to produce the final volume renders. Segmentation of cavities to produce virtual casts was also by region growing on a slice by slice basis.

A fifth specimen of *Aenigmachanna gollum*, 60.6 mm SL (KUFOS 2019.8.226), was cleared and double-stained^41^ to complement information obtained from the CT scans.

For the morphological analysis we studied cleared and double stained specimens of the following taxa of the order Labyrinthici: *Pristolepis fasciata* (MTD-F39837), *Badis corycaeus* (MTD-F39838), *Nandus nandus* (MTD-F39839), *Ctenopoma multispine* (MTD-F39840), *Sandelia bainsii* (MTD-F39841), *Belontia hasselti* (MTD-F39842), *Parachanna africana* (MTD-F39824), *P. obscura* (MTD-F39843), *Channa micropeltes* (MTD-F39844, MTD-F39845), *C. bankanensis* (MTD-F39846), *C. marulius* (MTD-F39847), *C. punctata* (MTD-F39825), *C. gachua* (MTD-F39848) and *C. bleheri* (MTD-F39849), in addition to *Aenigmachanna*. We scored 46 characters (see Supplementary Files) for the listed 15 taxa and conducted a parsimony analysis of the resulting data matrix using PAUP* v4.0a^42^. A heuristic search with a random-addition sequence (ten replicated) and tree bisection reconnection (TBR) branch swapping was conducted to find equally parsimonious trees, using unordered characters and assigning all characters equal weight. Node support was estimated using 1000 bootstrap replicates and character evolution was traced on the resulting phylogenetic trees using the DELTRAN option in PAUP* for selected characters in Fig. 5, but using the DELTRAN and ACCTRAN options for all characters in Supplementary Figs. S1 and S2.

### Molecular

To reconstruct the phylogenetic position of *Aenigmachanna* we assembled two different datasets with distinct taxon and gene sampling:

Dataset 1 consisted of 20 Channoidei (1 *Aenigmachanna*, 17 *Channa*, and 2 *Parachanna* species), 22 Anabantoidei representing all genera, and 2 members of the Labyrinthici families Badidae and Nandidae as outgroups. For this data set, the following marker sequences were used: (a) partial 12S and 16S; (b) coxI; (c) cytb; (d) the mitochondrial NADH dehydrogenase 2 (nd2) gene; (e) rag1; and the nuclear SH3 and PX domain-containing 3-like protein gene (sh3px3). The total alignment consisted of 5979 bp.

Dataset 2 consisted of 20 Channoidei (1 *Aenigmachanna*, 17 *Channa*, and 2 *Parachanna* species), 6 Anabantoidei representing all 3 families, and 1 member of the Labyrinthici family Nandidae as outgroup. This dataset consisted of the 13 protein coding genes from whole mitochondrial genomes and the final alignment consisted of 11,409 bp.

Our two datasets consisted of both newly determined sequences and sequences retrieved from Genbank (Supplementary Tables S1, S2). For the DNA sequences generated in India of the holotype and paratype of *Aenigmachanna gollum*, the following DNA extraction protocol was used: Gills were harvested from fresh specimen and were preserved in 100% ethanol. DNA was extracted using QIAamp DNA Mini Kit (Qiagen) following manufacturer’s instructions. Five gene fragments were sequenced including four mitochondrial and two nuclear gene fragments: (a) partial mitochondrial 12S rRNA gene, (b) partial mitochondrial 16S rRNA gene, (c) partial mitochondrial coxI, (d) complete mitochondrial cytb, (e) rag1, and (f) sh3px3. The nd2 was taken from the whole mt genome sequences (see below). Amplified DNA fragments were purified using the Wizard SV Gel PCR clean-up system (Promega) followed by Sanger sequencing by 1st BASE, Axil Scientific Pte Ltd, Singapore.

For the generation of the *Aenigmachanna* mt genomes DNA was isolated using a salting out protocol^43^. The mt genomes were amplified in their entirety using a long PCR technique as described in^44–46^. The PCR products were sent to Clevergene Biocorp Pvt Ltd (Bangalore, India) where the library preparation and further procedures were conducted for Illumina sequencing. The amplicons (there were two amplicons one ~ 7000 bp and the second ~ 12,000 bp) were fragmented using Covaris M-220 Focussed Ultrasonicator, to generate 300 bp fragments. These fragments were used for library preparation using NEB Next DNA II Library for Ilumina E7645S, using the manufacturers protocol. The QC passed libraries were sequenced using Illumina MiSeq to generate 2 × 301 paired end reads. The sequence data quality was checked using FastQC^47^ and MultiQC^48^ software. The data was checked for base call quality distribution, % bases above Q20, Q30, %GC, and sequencing adapter contamination. The sequence data was processed using Trim Galore to remove low quality bases and adapter sequences. The quality trimmed data was checked using FastQC and MultiQC. Quality passed reads were assembled using Higher Hierarchical Genome assembler (HGA)^49^. HGA produced three scaffolds with > 1000 bp length. The Norgal assembler was used to generate another assembly. Norgal (de Novo ORGAneLle extractor)^50^ is designed to assemble de novo mtDNA genome from de novo genome sequence data. Assembly was made with kmer range of 21–255 with interval of 28. Contig length threshold of 500 bp was set for the assembly. The circular genomes were annotated using MITOS web server^51^. There were 2 rRNA, 22 tRNA and 13 protein coding genes in each mt genome. A detailed analysis and characterization of the mt genome of *Aenigmachanna* is in preparation.

For newly determined sequences other than for *Aenigmachanna*, the protocols for DNA extraction, PCR primers and PCR conditions and sequencing by LGC Genomics, Berlin follow previous protocols^30,52–55^. For the sh3px3, the nested PCR protocol of Li et al*.*^56^ was used.

Chromatogram traces/raw reads were edited and assembled into contigs using Geneious v10.2.6^57^ and individual gene by gene datasets were complimented with sequences retrieved from GenBank prior to conducting an alignment with default settings using MAFFT v7.017^58^ as implemented in Geneious v10.2.6. The alignments of the protein coding genes were checked for frameshifts and premature stop codons. The individual gene by gene alignments were concatenated in Geneious v10.2.6 prior to phylogenetic analyses. Details of specimens used, datasets and GenBank accession numbers are given in Supplementary Table S1.

The optimal partitioning (for subsequent RAxML, and BEAST analysis of dataset 1) and substitution model scheme (for subsequent BEAST analysis of dataset 1) for the concatenated dataset were generated using PartitionFinder 1.0.1^59^ using initial partitions according to gene and codon position in case of protein coding genes. The setting model_selection = BIC and search = greedy was used for the different PartitionFinder runs (models = raxml and beast (dataset 1 only), respectively). The PartitionFinder results and subsequent partition strategies and models used for the different datasets are shown in Supplementary Table S3.

Maximum likelihood analyses was conducted with RAxML v7.3.4^60^, implementing the GTRGAMMA model for all partitions as identified by PartitionFinder under the − f a setting and 1000 bootstrap replicates. The three possible alternative topologies regarding the phylogenetic placement of *Aenigmachanna* among Channoidei were evaluated employing the approximately unbiased (AU^61^), Kishino–Hasegawa (KH^62^), and Shimodaira–Hasegawa (SH^63^) tests as implemented in CONSEL version 0.1k^64^ following^65^. The three alternative topologies were: (*Aenigmachanna* (*Parachanna*, *Channa*)), (*Parachanna* (*Aenigmachanna*, *Channa*)), and (*Channa* (*Aenigmachanna*, *Parachanna*)).

Divergence times for dataset 1 were estimated in BEAST v2.4.5 using an uncorrelated lognormal relaxed molecular clock. The Yule process was used as the speciation model and the partitions as defined by PartitionFinder. The options unlink substitution model, unlink clock model, and link all tree models were selected. The following priors were changed from their default value: clock rate (usld.mean) for the three genes were changed to Gamma (1, 1), initial = 1; and all p substitution parameters (GTR substitution parameters) were changed from Gamma to InverseGamma. We used the channid fossil^†^*Parachanna fayumensis*^66^ from the Jebel Qatrani Formation , Fayum Depression in Egypt , Late Eocene to Oligocene (about 35–33 mya) and the anabantoid fossil *Osphronemus* from the Sangkarewang Formation in Central Sumatra^67^, a formation dating back to the Early Oligocene to Late Eocene age (28.5–37.0 mya). Following Rüber et al*.*^30^ these two lognormal calibration priors from the fossil record were used: TMRCA *Parachanna* crown (offset 33.0; 37.0 Mya 95% soft upper bound; log mean = 0.1; log stdev = 0.8) and the TMRCA *Osphronemus* crown (offset 28.5; 35.0 Mya 95% soft upper bound; log mean = 0.23; log stdev = 1.0). Alternative calibrations were conducted assigning the *Parachanna* and *Osphromenus* fossils to their respective stem groups as in^30^. For a discussion on the Channoidei fossil record see^30^.

We used both, crown and stem calibrations because fossils are assigned to a branch based on the morphological characters they have. However, for calibrations fossils need to be assigned to a node. As each branch on a tree has two nodes that limit it, we calculated minimum ages for both options assigning it to the crown node and assigning it to the stem node. When the fossil is assigned to the crown node (our crown calibration) the calculated age for a specific lineage will be older than if the fossil is assigned to the stem node (our stem calibration). Calculating both options provides us with a better estimate of the ranges of minimum clade ages. The MCMC chain was run for 100 million iterations sampling every 10,000 steps and a conservative burnin of 20.000 steps. These settings were sufficient to ensure convergence and obtain ESS values > 200.

## Supplementary information

Supplementary Information.

## Data Availability

The DNA sequences generated and analysed for this project are available from Genbank and their accession numbers are provided in Supplementary Tables S1 and S2. The CT scan datasets generated and analysed during the current study are still being further analysed but are available upon reasonable request from the corresponding author.

## Code availability

This published work and the nomenclatural acts it contains have been registered in ZooBank, the proposed online registration system for the International Code of Zoological Nomenclature (ICZN). The ZooBankLSIDs (Life Science Identifiers) can be resolved and the associated information viewed through any standard web browser by appending the LSID to the prefix ‘https://zoobank.org/’. The LSIDs for this publication are urn:lsid:zoobank.org:pub:8E962B9B-15C0-49BA-A502-B40B2BDD5D0F (article), and urn:lsid:zoobank.org:act:BEC67F1C-923C-43B0-A09E-947D3727EA33 (Aenigmachannidae).
