# Supplementary material for: Aenigmachannidae, a new family of snakehead fishes (Teleostei: Channoidei) from subterranean waters of South India
Source: Sci Rep. 2020 Sep 30;10:16081. doi: 10.1038/s41598-020-73129-6 (PMC7527459; doi:10.1038/s41598-020-73129-6)
Supplement: Supplementary file 1 — Supplementary Information. [file 41598_2020_73129_MOESM1_ESM.pdf]

Electronic supplementary material for

Aenigmachannidae, a new family of snakehead fishes (Teleostei: Channoidei) from subterranean waters of South India

Ralf Britz[1,2], Neelesh Dahanukar[3,4], V.K. Anoop[5], Siby Philip[6], Brett Clark[7], Rajeev Raghavan[8] and Lukas Rüber[9,10]

[1]Senckenberg Natural History Collections Dresden, Museum of Zoology, DE-01109 Dresden, Germany. E-mail: ralf.britz@senckenberg.de.

[2]Department of Life Sciences, Natural History Museum, London, SW75BD, U.K.

[3]Indian Institute of Science Education and Research (IISER), Dr. Homi Bhabha Road, Pashan, Pune 411 008, India

[4]Zoo Outreach Organization, No. 12 Thiruvannamalai Nagar, Saravanampatti - Kalapatti Road, Coimbatore 641 035, India

[5]School of Ocean Science and Technology, Kerala University of Fisheries and Ocean Studies (KUFOS), Kochi 682 506, India

[6]Department of Zoology, Nirmalagiri College, Kannur, India

[7]Imaging and Analysis Centre, The Natural History Museum, London, SW7 5BD, United Kingdom.

[8]Department of Fisheries Resource Management, Kerala University of Fisheries and Ocean Studies (KUFOS), Kochi 682 506, India

[9]Naturhistorisches Museum Bern, 3005 Bern, Switzerland

[10]Aquatic Ecology and Evolution, Institute of Ecology and Evolution, University of Bern, 3012 Bern, Switzerland

Morphological characters

To analyse the phylogenetic position of *Aenigmachanna* in relation to the family Channidae and other Labyrinthici, we compiled a data matrix including 46 characters scored for the following 15 Taxa: the pristolepidid *Pristolepis fasciata*, the badid *Badis corycaeus*, the nandid *Nandus nandus*, the anabantid anabantoids *Sandelia bainsii* and *Ctenopoma multispine*, the osphronemid anabantoid *Belontia hasselti*, the channid channoids *Parachanna africana*, *P. obscura*, *Channa bankanensis*, *C. micropeltes*, *C. marulius*, *C. punctata*, *C. gachua*, *C. bleheri* and the aenigmachannid channoid *Aenigmachanna gollum*. We were unable to include the second species, *A. mahabali*, only known from the holotype, which was not available for further study. Given its overall very close resemblance to *A. gollum*, however, we do not expect it to differ in any significant way from what we report here for *A. gollum*. *Pristolepis* was chosen as outgroup. The following character matrix was analyzed in PAUP* (- represents inapplicable characters).

```
[                       0000000001111111111222222222233333333334444444]
[                       1234567890123456789012345678901234567890123456]
Aenigmachanna_gollum    10010-1-11011101000000---11000-00-000000100000
Parachanna_africana     10010-1111111011101111001100010011001000100000
Parachann_obscura       10010-111111101110111110010-0010011001000100000
Channa_micropeltes      10010-1111111011101111010100010011101011100000
Channa_bankanensis      10010-1111111011101111010100010010101001100000
Channa_marulius         10010-1111111011101111010100010111001011100000
Channa_punctata         10010-111110101110111110100-0010111001011100000
Channa_gachua           10010-111110101110111110100-0010111011011100000
Channa_bleheri          10010-1-111010111011110100-0010111011011100000
Belontia_hasselti       0110110000-11000-1000110011101100--00001001001
Ctenopoma_multispine    0110110000-11000-101011001111100-000001011111
Sandelia_bainsii        0110110000-11000-101011001111100-000001010100
Badis_corycaeus         0110100000-01100-00000---10000-00-000010010000
Nandus_nandus           0110100000-01100-00000---10000-00-000110011111
Pristolepis_fasciata    0110100000-00000-00000---10000-00-000110011111
```

The 46 morphological characters used for the PAUP* analysis are:

1. Nasal tubes: absent (0); present (1). All Channoidei including *Aenigmachanna* have the anterior nares located at the end of long nasal tubes. In all other Labyrinthici such nasal tubes are absent and the anterior naris is just an opening or is at the end of a short tube.

2. Scales: cycloid or crenate (0) transforming ctenoid (1). *Aenigmachanna* and all channids have scales covering their bodies that lack any cteni in the posterior field. The posterior margin of the scale is rounded and not well ossified, and the circuli of the scales are arranged in parallel and end in pointed tips some distance from the margin. All non channoid Labyrinthici have the typical percomorph transforming ctenoid scales on their body with the posterior field carrying series of cteni.

3. Dorsal-fin spines: absent (0), present (1). *Aenigmachanna* and all channids have only soft (segmented) fin rays in their dorsal fin, fin spines are missing. All other Labyrinthici studied here have fin spines *Badis* (17), *Nandus* (13), *Pristolepis* (13), *Sandelia* (15), *Ctenopoma* (17), *Belontia* (18).

4. Dorsal-fin soft (segmented) rays: fewer than 20 (0); more than 20 (1). *Aenigmachanna* and all channids have more than 20 rays: *Aenigmachanna gollum* (51-56), *Parachanna africana* (47), *P. obscura* (42), *Channa bankanensis* (39), *C. micropeltes* (46), *C. marulius* (55), *C. punctata* (30), *C. gachua* (37), *C. bleheri* (35). All other Labyrinthici have fewer than 20 rays: *Pristolepis* (16), *Nandus* (13), *Badis* (10), *Sandelia* (9), *Ctenopoma*, (9), *Belontia* (12).

5. Anal-fin spines: absent (0), present (1). *Aenigmachanna* and all channids have only soft (segmented) fin rays in their anal fin; fin spines are missing. All other Labyrinthici have several anal-fin spines (see character 6).

6. Anal-fin spines: three (0); more than three (1). *Pristolepis*, *Nandus* and *Badis* have three anal-fin spines. All anabantoids have more than three anal-fin spines: *Sandelia* (7), *Ctenopoma* (9), *Belontia* (16)

7. Anal-fin soft (segmented) rays: fewer than 20 (0); more than 20 (1). *Aenigmachanna* and all channids have more than 20 soft anal-fin rays: *Aenigmachanna gollum* (40-44), *Parachanna africana* (32), *P. obscura* (30), *Channa bankanensis* (29), *C. micropeltes* (30), *C. marulius* (33), *C. punctata* (22), *C. gachua* (25), *C. bleheri* (24). All other Labyrinthici have fewer than 20 soft rays: *Pristolepis* (9), *Nandus* (8), *Badis* (8), *Sandelia* (9), *Ctenopoma*, (9), *Belontia* (12).

8. Pelvic fin spine: present (0), absent (1). In *Aenigmachanna* and *Channa bleheri* the pelvic fin is missing. All Labyrinthici except channids have a pelvic-fin spine. The first pelvic-fin ray in channids is a segmented, unbranched soft ray.

9. Vertebrae: fewer than 35 (0), more than 35 (1). *Aenigmachanna* and all channids have elongate bodies with more than 35 vertebrae. *Aenigmachanna gollum* (29-32+29-31=58-63), *Channa marulius* (48+5=53), *Channa punctata* (33+4=37) *Channa micropeltes* (51+5=56), *Channa bankanensis* (39+4=43), *Parachanna africana* (41+4=45) *Channa bleheri* (39+5=44), *Channa gachua* (37+5=42), *Parachanna obscura* (37+5=42). All other Labyrinthici have by far fewer vertebrae: *Badis* (15+13=28), *Nandus* (12+11=23), *Pristolepis* (13+12=25), *Sandelia* (12+18=30), *Ctenopoma* (12+19=31), *Belontia* (10+18=28)

10. Postanal abdominal vertebrae: absent (0); 10 and more (1). In all Labyrinthici except *Aenigmachanna* and channids the anus is situated at the level of the first caudal vertebra or in front of it, there are no postanal abdominal vertebrae. In *Aenigmachanna* and channids the abdominal cavity extends posteriorly beyond the anus and there are ten or more abdominal vertebrae behind the anus.

11. Postanal abdominal vertebrae: 10-12(0); more than 12 (1). *Aenigmachanna* and all channids show an abdominalisation of their postanal vertebral column. In *Aenigmachanna* the first ten postanal vertebrae are abdominalised but all more posterior vertebrae are caudal vertebrae. In channids, all postanal vertebrae are abdominalised except the posterior most 4-6, which are caudal vertebrae. There is no abdominalisation of postanal vertebrae in non-channoid Labyrinthici.

12. Number of epurals: 1(0) or 2(1). *Pristolepis, Nandus* and Badis have only a single epural, all other Labyrinthici have two epurals.

13. Full neural spine on PU2: absent (0); present (1). There is a full neural spine on the second preural centrum in all Labyrinthici except *Pristolepis*, which has a shortened neural spine on PU2.

14. Bifurcated haemal spine of PU2: absent (0), present (1). Among Labyrinthici, only *Badis, Nandus,* and *Aenigmachanna* have a bifurcated haemal spine. This character was previously used as a putative synapomorphy of Nandidae + Badidae by Kullander & Britz (2002) and Collins et al. (2015).

15. Day's bone: absent (0); present (1). All channids have an autogenous independently chondrifying ossicle, Day's bone, between the haemal spines of PU2 and PU3. Day's bone is absent in all other Labyrinthici.

16. Swim bladder extension single: absent (0); present (1). In *Aenigmachanna* and all channids the posterior swim bladder extension is single. All anabantoids have paired extensions of the swim bladder and *Pristolepis*, *Nandus* and *Badis* have no extensions.

17. Single swim bladder extension reaching: to postanal vertebra 8 (0); to parhypural (1). The single posterior swim bladder extension reaches posteanal vertebra no. 8 in *Aenigmachanna*, but reaches farther posteriorly in channids up to the parhypural.

18. Posterior swim bladder extension paired: absent (0); present (1). All anabantoids have paired extensions of the swim bladder running on each side of the hemal spines up

to the parhypural. *Aenigmachanna* and channids have a single extension. *Pristolepis*, *Nandus* and *Badis* have no extensions.

19. Autosphenotic: does not carry lateral line canal (0); carries lateral line canal (1). In all Channidae the autosphenotic carries the lateral line canal between the frontal and the pterotic. In all other Labyrinthici including *Aenigmachanna* the autosphenotic does not carry a lateral-line canal. In some anabantids the autosphenotic may approach the lateral-line canal in the frontal and pterotic, but is not traversed by the canal as in channids.

20. Autosphenotic: not forming part of skull roof (0); forming part of skull roof (1). In all channids and in *Sandelia* and *Ctenopoma*, the autosphenotic reaches the skull roof and forms a small (*Sandelia*, *Ctenopoma*) or larger (channids) part of its lateral margin. In all other Labyrinthici including Aenigmachanna the autosphenotic does not contribute to the skull roof and is situated on the lateral side of the neurocranium and covered dorsally by frontal and pterotic.

21. Otic bulla for sacculith: formed by prootic and exoccipital (0); formed mostly by prootic (1). In all channids, the prootic forms the majority of the bulla that houses the sacculith. In all other Labyrinthici including *Aenigmachanna* both prootic and exoccipital contribute equally to formation of the bulla.

22. Basioccipital articular processes: absent (0); present (1). In all channids and anabantoids, the basioccipital has paired articular processes, flanges or crests with which the upper pharyngeal jaws articulate via PB2+3. Such paired processes are absent in all other Labyrinthici including *Aenigmachanna*.

23. Basioccipital articulation a stout condyle: absent (0), present (1). In all anabantoids the basioccipital articulation with which the upper pharyngeal jaws articulate is formed by a pair of stout condyles approaching each other closely in the ventral midline. The articular surface in channids is different from that in anabantoids.

24. Basioccipital articulation an oval flange: absent (0); present (1). In species of the genus *Channa* the articular surface on the basioccipital is developed as an oval flange rather than a stout condyle as in anabantoids or an elongate crest as in *Parachanna*.

25. Basioccipital articulation an elongate, narrow crest: present (0); absent (1). The paired basioccipital articulations with the upper pharyngeal jaws are developed as elongate narrow horizontal crests running along the lateral margins of the basioccipital in *Parachanna*. Or these flanges can be circular to oval pads, as in the other channids. In anabantoids, the processes are stout and ventrally directed and approach each other closely in the ventral midline.

26. Parasphenoid teeth: present (0); absent (1). All Labyrinthici have parasphenoid teeth, except *Parachanna obscura*, *Channa punctata*, *C. gachua*, and *C. bleheri*.

27. Parasphenoid tooth patch: with small conical teeth only (0); with large anteriorly curved teeth (1). Most channids and *Badis*, *Nandus*, and *Pristolepis* have small, sometimes numerous, conical teeth on the parasphenoid, except in our specimens of *Parachanna obscura*, *Channa punctata*, *C. gachua* and *C. bleheri*, in which the parasphenoid is toothless. All anabantoids and *Aenigmachanna* have large anteriorly curved teeth on the parasphenoid.

28. Pharyngeal process of parasphenoid. In all Anabantoidei, the parasphenoid has a deep vertical crest-like extension, the pharyngeal process. Such an extension is lacking in all other Labyrinthici.

29. Transverse parasphenoid process: absent (0); present (1). *Sandelia* and *Ctenopoma* have toothed transverse processes on the parasphenoid. Such processes are lacking in the other Labyrinthici.

30. Suprabranchial cavity and labyrinth organ: absent (0); present (1). All anabantoids and all channids have paired suprabranchial cavities, which house the labyrinth organ and into which they swallow air to complement their oxygen supply from the gills. All

other Labyrinthici including *Aenigmachanna* do not have a suprabranchial cavity or a labyrinth organ.

31. Suprabranchial cavity: openly communicating with buccal cavity (0); separated almost completely and only communicating via small opening guarded by modified gill raker (1). In channids the suprabranchial cavity communicates openly with the buccal cavity, resulting in air escaping from the chamber when they are suction feeding. In anabantoids, communication between the suprabranchial cavity and the buccal cavity is greatly narrowed to a small circular opening, which is guarded by a modified gill raker that serves as a valve.

32. Hyomandibular bony medial process: absent (0); present (1). The hyomandibular of some species of *Channa* (*C. marulius*, *C. punctata*, *C. gachua*, *C. bleheri*) has a bony medial process that projects into the suprabranchial and buccal cavities forming a vertical portioning wall. Such a bony process is absent in other channids and in other Labyrinthici including *Aenigmachanna*.

33. Metapterygoid ascending process: absent (0); present (1). All channids have an ascending process on the metapterygoid, which may or may not reach the frontal. All other Labyrinthici including *Aenigmachanna* lack an ascending process.

34. Ascending process of metapterygoid articulates with frontal: absent (0); present (1). In *Parachanna* and *Channa* except *C. bankanensis*, the metapterygoid has a long anterodorsal process that articulates with the ventral face of the frontal. In *Channa bankanensis* the process is present but shorter and does not reach the frontal. All other Labyrinthici including *Aenigmachanna* lack such a process.

35. Palatine, if toothed: with small teeth of similar size only (0);additionally with caniniform teeth (1). In all Labyrinthici with palatine teeth there is a series of small conical teeth, but in *Channa micropeltes* and *C. bankanensis* the palatine also carries several large caniniform teeth.

36. Ectopterygoid: present (0); absent (1). All Labyrinthici have an ectopterygoid, but this bone is absent in *C. gachua* and *C. bleheri*, in which the entopterygoid has expanded into its position.

37. Caniniform teeth along dentary: absent (0); present (1). In all channids the dentary has a series of large caniniform teeth along the dentary in addition to small conical teeth. In all other Labyrinthici, including *Aenigmachanna*, the dentary has small conical teeth and if caniniform teeth are present they are restricted to the anterior part of the dentary usually near the symphysis.

38. Basihyal teeth: absent (0); present (1). The basihyhal of *Nandus* and *Pristolepis* bears a number of teeth, in all other Labyrinthici including *Aenigmachanna*, the basihyal is not toothed.

39. HB3 teeth: absent (0); present (1). Hypobranchial 3 is toothed in *Pristolepis*, *Nandus* and *Badis* and all species of *Channa* except *C. bankanensis*. It is also toothless in *Parachanna*, *Aenigmachanna* and all anabantoids.

40. Epibranchial 1: not expanded by membrane bone (0); expanded by membrane bone (1). In all species of *Channa* and all Anabantoidei epibranchial 1 has a membrane bone extension giving the bone a laminar appearance. In all other Labyrinthici, including *Parachanna* and *Aenigmachanna* the epibranchial is an ossified rod and lacks the lamina of membrane bone.

41. Branchiostegal rays: 6 (0); 5 (1). *Aenigmachanna* and all Channidae have five branchiostegal rays, but all other Labyrinthici have six.

42. Opercular spination: absent (0); present (1). *Aenigmachanna*, all channids, and *Belontia* lack the opercular spination that is present in the other Labyrinthici.

43. Preopercular spination: absent (0); present (1). Most Labyrinthici lack the spination on the preopercular that characterises *Pristolepis*, *Nandus*, *Ctenopoma* and *Belontia*.

44. Subopercular spination: absent (0); present (1). Most Labyrinthici lack the spination of the subopercular that characterises *Pristolepis*, *Nandus*, *Sandelia*, and *Ctenopoma*.

45. Interopercular spination: absent (0); present (1). Most Labyrinthici lack the spination of the interopercular that characterises *Pristolepis*, *Nandus* and *Ctenopoma*.

46. Lachrymal spination: absent (0); present (1) Most Labyrinthici lack the spination of the lachrymal that characterises *Pristolepis*, *Nandus*, *Ctenopoma* and *Belontia*.

Supplementary figures

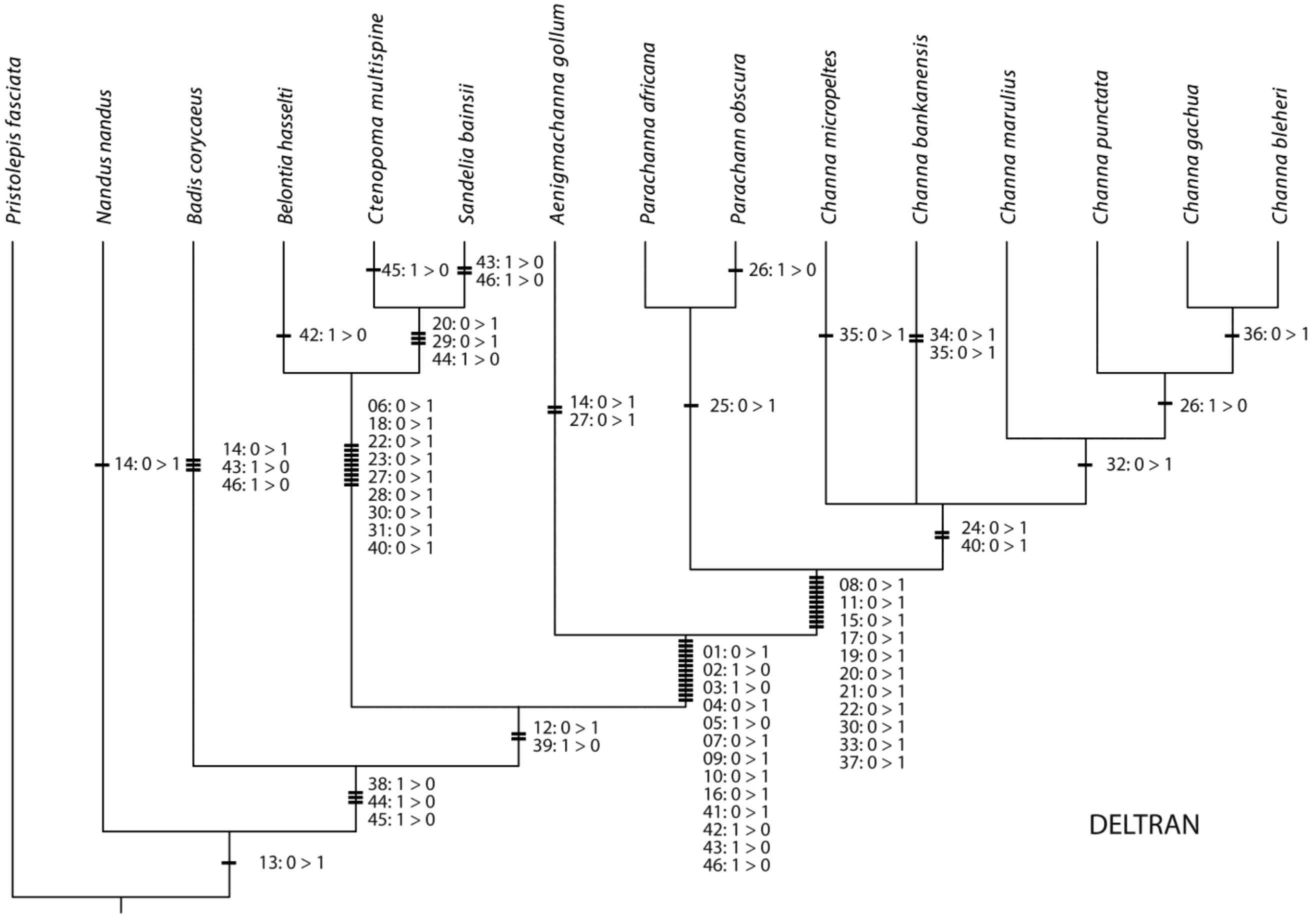

Supplementary Figure S1. Phylogenetic tree of morphological dataset showing character state changes using DELTRAN.

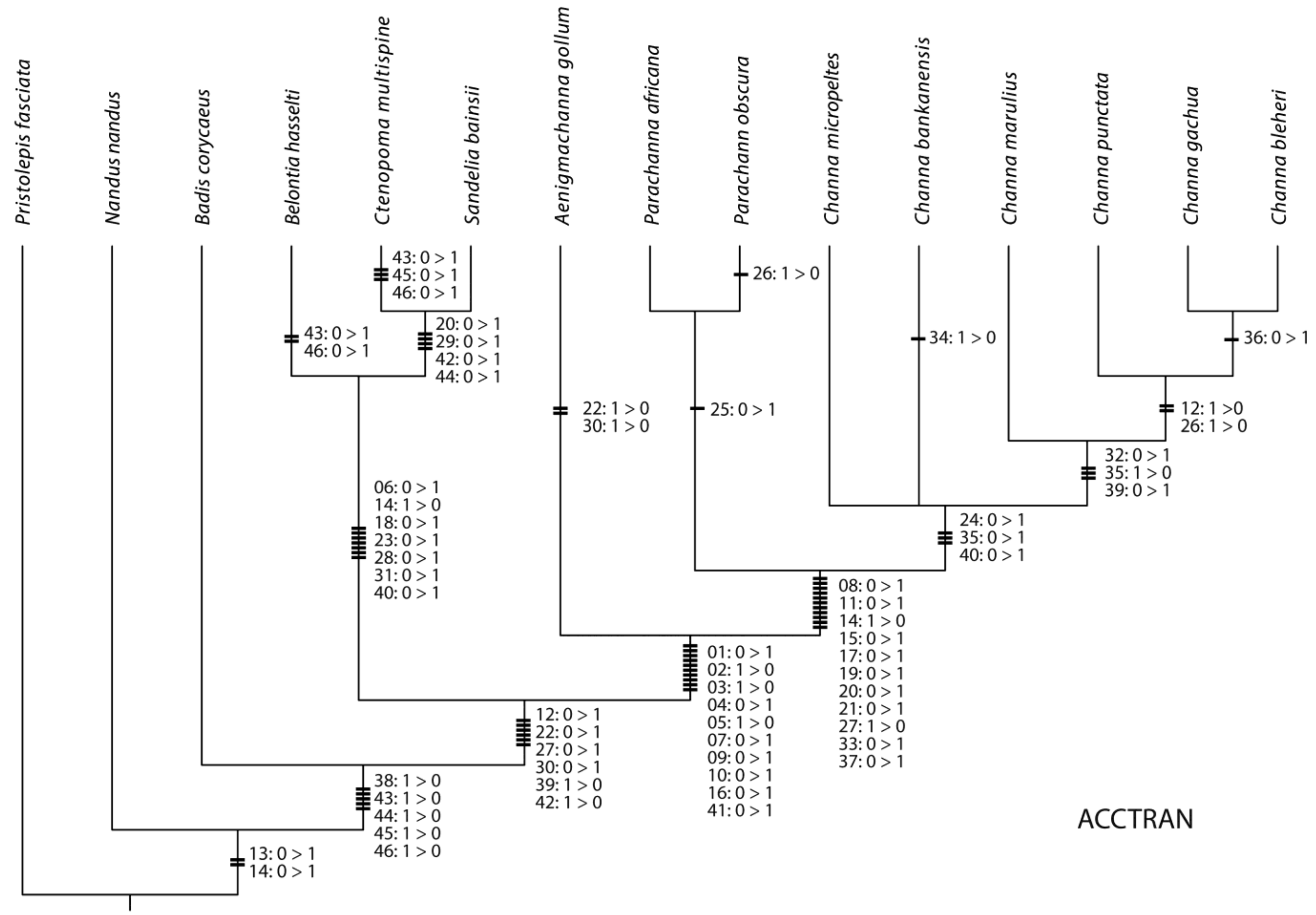

Supplementary Figure S2. Phylogenetic tree of morphological dataset showing character state changes using ACCTRAN

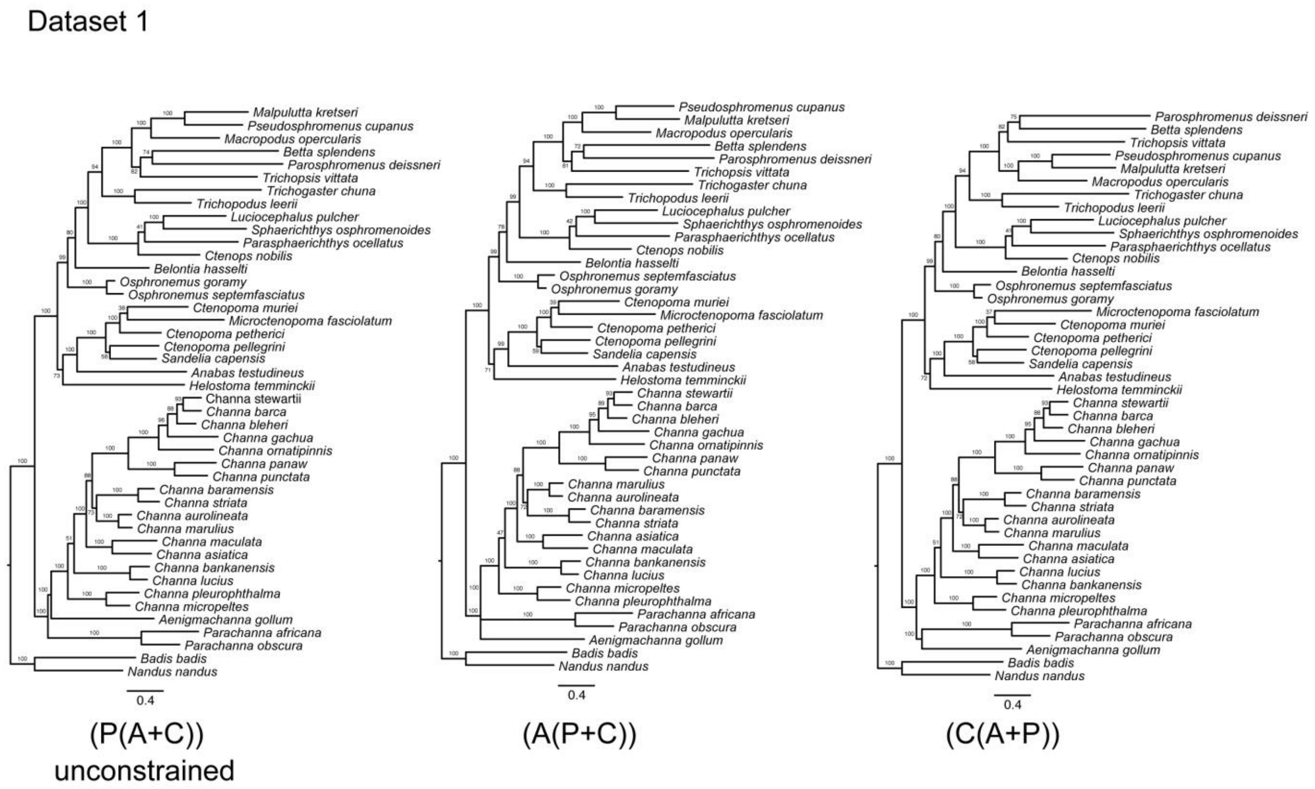

Supplementary Figure S3. Unconstrained and two constrained ML trees with bootstrap values based on data set 1.

Dataset 2

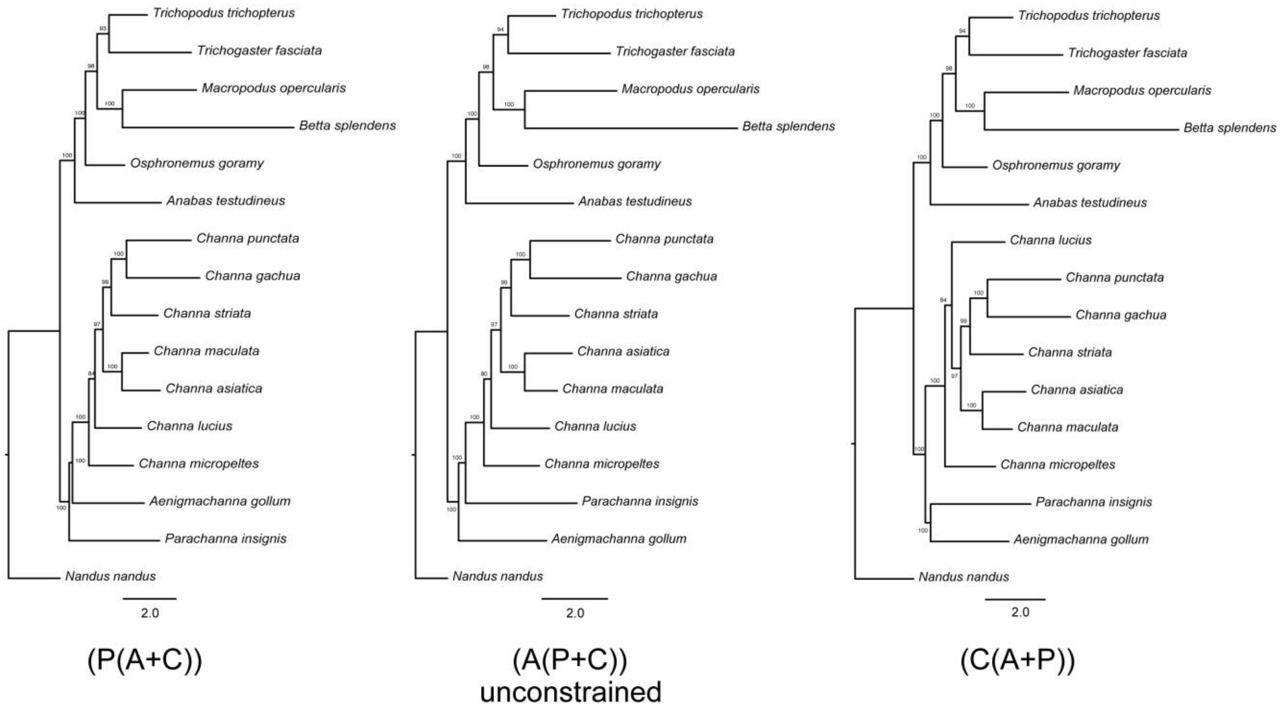

Supplementary Figure S4. Unconstrained and two constrained ML trees with bootstrap values based on data set 2.

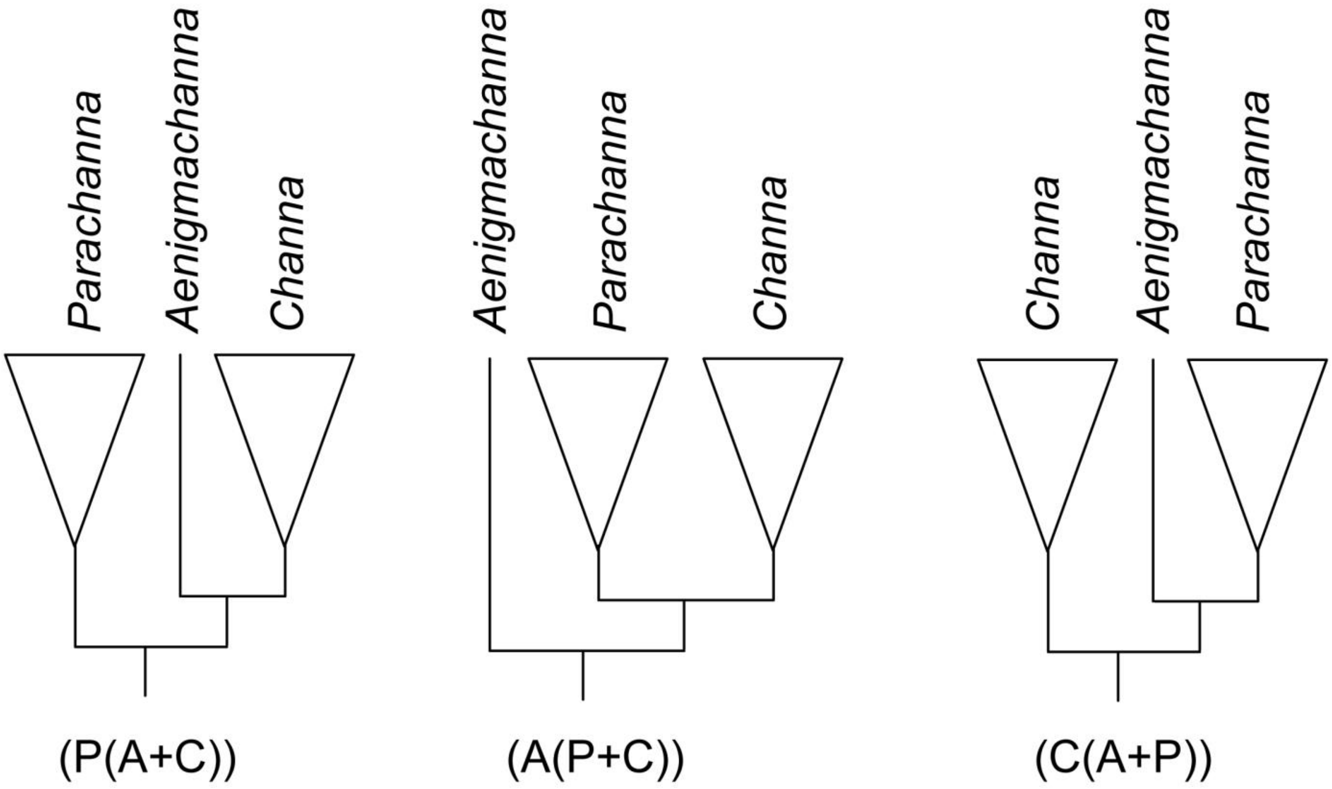

	-ln *L*	*P* (AU)	*P* (KH)	*P* (SH)
Dataset 1				
(P(A+C)) unconstrained	69756.859	0.683	0.645	0.749
(A(P+C))	69759.829	0.073	0.161	0.233
(C(A+P))	69758.198	0.409	0.355	0.456
Dataset 2				
(P(A+C))	99442.123	0.166	0.145	0.194
(A(P+C)) unconstrained	99433.945	0.871	0.846	0.911
(C(A+P))	99441.729	0.185	0.154	0.208

Supplementary Figure S5. Illustration of the three alternative topologies tested for data sets 1-2. Log-likelihoods and *P* values of AU, KH, and SH tests for the alternative topologies evaluated.

Species	GenBank accession				
	rRNA	cox1	cytb	nd2	rag1
Aenigmachanna gollum 1 (not used)	MT936903	MK798108	MT928178	--	MT928180
Aenigmachanna gollum 2	MT936902	MK798107	MT928177	MT670416	MT928179
Channa asiatica	MN057420	MF496666	MN056979	KJ930190	MN057198
Channa aurolineata	MN057428	MF496843	MN056987	n/a	MN057206
Channa bankanensis	MN057436	MF496676	MN056995	AB196275	MN057214
Channa baramensis	MN057438	MF496695	MN056997	n/a	MN057216
Channa barca	MN057441	MF496699	MN057000	AB196278	MN057219
Channa bleheri	AY763724	EU924638	AY763770	AB196273	LT577443
Channa gachua	MN057455	MF496725	MN057014	AB196274	MN057233
Channa lucius	MN057516	MF496817	MN057075	MF804538	MN057294
Channa maculata	MN057523	MF496835	MN057082	KC823606	MN057301
Channa marulius	MN057530	MF496844	MN057089	KF420268	MN057308
Channa micropeltes	MN057546	MF496863	MN057105	KX129904	MN057324
Channa ornatipinnis	MN057553	MF496871	MN057111	n/a	MN057331
Channa panaw	MN057557	MF496877	MN057115	AB196267	MN057335
Channa pleurophthalma	MN057560	MF496881	MN057118	AB196269	MN057338
Channa punctata	MN057568	MF496887	MN057126	AB196280	MN057346
Channa stewartii	MN057594	MF496931	MN057152	n/a	MN057372
Channa striata	MN057596	MF496933	MN057154	KX177965	MN057374
Parachanna africana	MN057625	MF496972	MN057183	AB196264	MN057403
Parachanna obscura	MN057630	MF496978	MN057188	AB196265	MN057408
Anabas testudineus	AY763681	HQ682664	AY763727	KT001153	AY763773
Belontia hasselti	AY763697	KM213043	AY763743	n/a	AY763780
Betta splendens	AF519650	GQ911736	AF519689	KR527219	AF519728
Ctenopoma muriei	AY763684	n/a	AY763730	n/a	AY763774
Ctenopoma pellegrini	AY763689	n/a	AY763735	n/a	AY763776
Ctenopoma petherici	AY763687	n/a	AY763733	n/a	AY763775
Ctenops nobilis	AY763702	TBA3	AY763748	n/a	AY763781
Helostoma temminckii	AY763696	KM213051	AY763742	AB861523	AY763779
Luciocephalus pulcher	AY763703	TBA4	AY763749	n/a	AY763782
Macropodus opercularis	AF519659	KU568904	AF519698	n/a	AF519737
Malpulutta kretseri	AF519661	n/a	AF519700	n/a	AF519739
Microctenopoma fasciolatum	AY763692	n/a	AY763738	n/a	AY763777
Osphronemus goramy	AY763722	KU692698	AY763768	KU984978	AY763785
Osphronemus septemfasciatus	AY763723	n/a	AY763769	n/a	MN057412
Parasphaerichthys ocellatus	AY763706	TBA5	AY763752	n/a	AY763783
Parosphromenus deissneri	AF519662	TBA6	AF519701	n/a	AF519740
Pseudosphromenus cupanus	MN057634	KC774665	MN057192	n/a	MN057413
Sandelia capensis	AY763695	n/a	AY763741	n/a	AY763778
Sphaerichthys osphromenoides	AY763708	TBA7	AY763754	n/a	AY763784
Trichogaster chuna	AF519657	KJ936825	AF519696	n/a	AF519735
Trichopodus leerii	AF519656	TBA8	AF519695	KR029983	AF519734
Trichopsis vittata	AF519658	GQ911985	AF519697	AB196285	AF519736
Badis badis	MN057635	AY662746	AY330939	n/a	MN057414
Nandus nandus	MN057636	JN815306	AY330963	AP017449	AY330979

Supplementary Table S1. Table of specimens and genes used in data set 1 together with their GenBank accession numbers.

Species	GenBank accession
Aenigmachanna gollum 2	MT670416
Channa asiatica	KJ930190
Channa gachua	NC036948 or MF924390
Channa lucius	MF804538
Channa maculata	KC823606
Channa micropeltes	KX129904.1
Channa punctata	NC 042213
Channa striata	KX177965
Parachanna insignis	AP006042
Anabas testudineus	KJ808811
Betta splendens	AB571120
Macropodus opercularis	KP234027 or KM588227
Osphronemus goramy	KU984978
Trichogaster fasciata	KP301136
Trichopodus trichopterus	KP100265 or LC026155
Nandus nandus	AP017449

Supplementary Table S2. Table of specimens and genes used in data set 2 together with their GenBank accession numbers.

Dataset	Partition	Modell	Position in alignment
Dataset 1 (RAxML)	1) rRNA	GTR+G	1-972
	2) cox1 1st, cytb 1st	GTR+G	973-1626\3, 1627-2754\3
	3) snx 2nd, cox1 2nd, rag 2nd	GTR+G	974-1626\3, 3797-5289\3, 5291-5979\3
	4) cox1 3rd	GTR+G	975-1626\3
	5) nd2 2nd, cytb 2nd	GTR+G	1628-2754\3, 2756-3795\3
	6) nd2 3rd, cytb 3rd	GTR+G	1629-2754\3, 2757-3795\3
	7) nd2 1st	GTR+G	2755-3795\3
	8) snx 1st, rag 1st	GTR+G	3796-5289\3, 5290-5979\3
	9) snx 3rd, rag 3rd	GTR+G	3798-5289\3, 5292-5979\3
Dataset 1 (Beast)	1) rRNA	GTR+I+G	1-972
	2) cox1 1st	TrNef+I+G	973-1626\3
	3) cox1 2nd	TrN+I	974-1626\3
	4) cox1 3rd	TrN+I+G	975-1626\3
	5) cytb 1st	SYM+I+G	1627-2754\3
	6) cytb 2nd, nd2 2nd	HKY+I+G	1628-2754\3, 2756-3795\3
	7) cytb 3rd, nd2 3rd	GTR+I+G	1629-2754\3, 2757-3795\3
	8) nd2 1st	GTR+I+G	2755-3795\3
	9) rag 1st	K80+G	3796-5289\3
	10) rag 2nd, snx 1st, snx 2nd	HKY+I+G	3797-5289\3, 5290-5979\3, 5291-5979\3
	11) rag 3rd, snx 3rd	GTR+G	3798-5289\3, 5292-5979\3
Dataset 2 (RAxML)	1) atp6 1st, cox2 1st, cytb 1st, nd1 1st, nd3 1st, nd4 1st, nd4l 1st	GTR+G	1-681\3, 2395-3087\3, 3871-5007\3, 5008-5979\3, 7024-7374\3, 7375-8757\3, 11116-11409\3
	2) atp6 2nd, cox1 2nd, cox2 3rd, cox3 2nd, cytb 2nd, nd1 2nd, nd3 2nd, nd4 2nd, nd4l 2nd	GTR+G	2-681\3, 848-2394\3, 2396-3087\3, 3089-3870\3, 3872-5007\3, 5009-5979\3, 7025-7374\3, 7376-8757\3, 11117-11409\3
	3) atp6 3rd, atp8 3rd, cox1 3rd, cox2 2nd	GTR+G	3-681\3, 684-846\3, 849-2394\3, 2397-3087\3
	4) atp8 1st, nd2 1st, nd5 1st	GTR+G	682-846\3, 5980-7023\3, 8758-10596\3
	5) atp8 2nd, nd2 2nd, nd5 2nd, nd6 2nd	GTR+G	683-846\3, 5981-7023\3, 8759-10596\3, 10598-11115\3
	6) cox1 1st, cox3 1st	GTR+G	847-2394\3, 3088-3870\3
	7) cox3 3rd, cytb 3rd, nd1 3rd, nd2 3rd, nd3 3rd, nd4 3rd, nd4l 3rd, nd5 3rd	GTR+G	3090-3870\3, 3873-5007\3, 5010-5979\3, 5982-7023\3, 7026-7374\3, 7377-8757\3, 8760-10596\3, 11118-11409\3
	8) nd6 1st	GTR+G	10597-11115\3
	9) nd6 3rd	GTR+G	10599-11115\3

Supplementary Table S3. Results of Partitionfinder for ML analyses of datasets 1-2. Partitionfinder results for subsequent BEAST analysis of dataset 1 are also shown.
